# Supplementary material for: A UMLS-based spell checker for natural language processing in vaccine safety
Source: BMC Med Inform Decis Mak. 2007 Feb 12;7:3. doi: 10.1186/1472-6947-7-3 (PMC1805499; doi:10.1186/1472-6947-7-3)
Supplement: Additional file 13 — RAP application directory. Sets up directory for RAP (RDF application for PHP) source code [file 1472-6947-7-3-S13.gz › rap/doc/phpdoc/__filesource/fsource_default__syntaxSyntaxRDF.php.html]

xml version="1.0" encoding="iso-8859-1"?


File Source for SyntaxRDF.php


# Source for file SyntaxRDF.php

Documentation is available at SyntaxRDF.php

```
1. <?PHP
2. // ----------------------------------------------------------------------------------
3. // Syntax RDF
4. // ----------------------------------------------------------------------------------
5. //
6. // Description               : Syntax RDF package
7. //
8. // History:
9. // 08-21-2004                : Initial version.
10. //
11. // Author: Tobias Gau�    <tobias.gauss@web.de>
12. //
13. // ----------------------------------------------------------------------------------


16. // Include Syntax classes

18. require_once( RDFAPI_INCLUDE_DIR . 'syntax/RdfParser.php' );
19. require_once( RDFAPI_INCLUDE_DIR . 'syntax/RdfSerializer.php' );
20. require_once( RDFAPI_INCLUDE_DIR . 'syntax/NTripleSerializer.php' );
21. ?>
```

Documentation generated on Fri, 17 Dec 2004 16:18:13 +0100 by phpDocumentor 1.3.0RC3
